# Perceived Credibility of Health News and Its Relationship with Trust in Physicians and the Health Care System

**DOI:** 10.3390/healthcare14101389

**Published:** 2026-05-19

**Authors:** Erhan Dağ, Yaşar Demir, Mustafa Nal, Ekrem Sevim, Sevilay Güler, Gülfer Bektaş

**Affiliations:** 1 Gediz Health Services Vocational School, Kutahya Health Sciences University, Kutahya 43100, Türkiye; erhan.dag@ksbu.edu.tr; 2 Department of Statistics, Samsun City Hospital, Samsun 55090, Türkiye; yasar.demir1@saglik.gov.tr; 3 Department of Healthcare Management, Faculty of Health Sciences, Kutahya Health Sciences University, Kutahya 43100, Türkiye; mustafa.nal@ksbu.edu.tr; 4 Department of Healthcare Management, Faculty of Health Sciences, Bandirma Onyedi Eylul University, Balıkesir 10200, Türkiye; esevim@bandirma.edu.tr; 5 Department of Quality Coordination Office, Yalova University, Yalova 77100, Türkiye; sevilay.guler@yalova.edu.tr; 6 Department of Healthcare Management, Faculty of Health Sciences, Acibadem Mehmet Ali Aydinlar University, Istanbul 34752, Türkiye; 7 Department of Healthcare Management, Graduate School of Health Sciences, Acibadem Mehmet Ali Aydinlar University, Istanbul 34752, Türkiye

**Keywords:** trust, physicians, health news, healthcare, distrust, public health, digital health literacy

## Abstract

**Background**: This study fills a gap in the literature by examining how perceptions of health news are associated with interpersonal trust in physicians and institutional distrust in the healthcare systems, particularly in the Türkiye context. While previous studies have examined trust in physicians and distrust in healthcare systems separately using different independent variables, evidence remains limited on the simultaneous association between health news perception and both trust in physicians and distrust in the healthcare system, particularly in the Türkiye context and using validated measurement tools. Therefore, the main objective of this study is to examine the association between health news perception, trust in physicians, and distrust in the healthcare system. **Methods**: The population of this cross-sectional study consisted of individuals aged 18–60 residing in the central district of Kutahya. The study data were collected face-to-face using a two-part questionnaire. A total of 719 completed questionnaires were analyzed. **Results**: 58% of the participants in the study were aged 41 and above. Commercial concerns and advertising, consumption promotion, behavioral change, health behavior exploitation, and trust in health news were positively associated with distrust in the healthcare system (β = 0.119, β = 0.196, β = 0.054, β = 0.061, β = 0.046; *p* < 0.01, *p* < 0.05). The model explained 22.4% of the variance (R^2^ = 0.224). Commercial concerns and advertising, consumption promotion, behavioral change, health behavior exploitation, and trust in health news were negatively associated with trust in physicians (β = −0.221, β = −0.014, β = −0.014, β = −0.127, β = −0.211; *p* < 0.05, *p* < 0.01). The model explained 25.3% of the variance (R^2^ = 0.253). **Conclusions**: The study found that health news perception was associated with trust in physicians and distrust in the healthcare system. Therefore, understanding the associations between health news perception, trust in physicians, and distrust in the healthcare system is important for improving individual and public health. For this reason, it is of great importance to raise the level of health and digital health literacy in society through policies developed under the leadership of public health experts.

## 1. Introduction

Trust is a fundamental element in the effectiveness and quality of healthcare. The patient-physician relationship and the patient’s confidence in the healthcare system are pivotal in determining treatment adherence, the decision to seek healthcare, and the patient’s overall health outcomes. These dimensions are usually conceptualized as interpersonal and institutional trust. Interpersonal trust denotes confidence in individual physicians within the patient–physician relationship [1], whereas institutional trust or distrust involves broader evaluations of the healthcare system and its organizations [2,3,4]. These forms of trust are related; they operate at different levels. Health news perception may influence both patients’ views of physicians and their wider judgments about the healthcare system. Therefore, examining both outcomes together provides a stronger theoretical basis for understanding trust-related perceptions in healthcare. However, the credibility of health information in the media can be influenced by a variety of factors, both positive and negative. In this context, the content, presentation, and perception of health news are critical factors in shaping the public’s trust in health professionals and the health system [5,6].

Media and the Internet have become central sources of health information and can influence public perceptions of healthcare and have assumed a prominent role in numerous sectors, including healthcare, with their capacity to modify societal perceptions and attitudes [7]. Media effects theories suggest that media not only disseminate information but also shape public judgments about health-related issues. Specifically, the pervasive utilization of mobile Internet in recent years has amplified the scope and intensity of this impact. Accordingly, the growing use of mobile Internet has made its role in healthcare trust an important research issue [8].

Previous research generally examines trust in physicians at two levels: interpersonal trust in the patient–physician relationship and broader institutional trust in healthcare [8]. The initial study examines the patient-physician trust relationship at the individual level. This approach underscores the significance of individual factors in the establishment of trust by emphasizing the characteristics of both the patient and the treating physician [9]. In this context, factors such as patients’ honesty during the examination process, physicians’ efforts to protect patients’ privacy, and communication skills between the parties become salient [10,11]. This line of research emphasizes trust as a direct result of the quality of interpersonal interaction and communication. The second approach is characterized by its societal perspective, examining the concept of trust in physicians or the health care system in general [8]. These studies examine macro-level factors, including social structure, cultural dynamics, and intergroup differences in perception [2]. Additionally, they assess the impact of legislation and health policy [12,13]. In this framework, the media emerges as an important external actor influencing the formation of collective trust. Research at both levels frequently underscores the pivotal function of the media in shaping public trust in physicians and the healthcare system. Indeed, research has demonstrated that media content, particularly television programming, has the potential to influence the development of negative attitudes towards physicians. This phenomenon is associated with the increasingly negative portrayal of physicians in the media. A multitude of studies have demonstrated that unfavorable television news reports on medical issues have the capacity to diminish the public’s confidence in healthcare professionals and the healthcare system [14,15].

This phenomenon, influenced by the mass media, significantly impacts the public’s perception of physicians and the healthcare system. Extant literature suggests that this influence is often deleterious. Furthermore, the presence of selective journalism or fictional portrayals that do not accurately reflect reality in the media has been demonstrated to exert a substantial influence on individuals’ health information-seeking behaviors [16,17,18]. Such portrayals may foster unrealistic expectations of healthcare. Television series frequently contain misrepresentations of the healthcare system and healthcare professionals. Patients exposed to such misleading information may develop unrealistic expectations during communication with healthcare providers, which can trigger feelings of distrust toward healthcare personnel and even violence [19,20,21].

Health-related news media have garnered the attention of academic circles due to their impact on the public’s trust in medical professionals and the healthcare system. Consequently, there has been a marked increase in the number of studies addressing this subject. Recent technological advancements and the increasing use of the Internet and social media have prompted scholars to examine the impact of these platforms on media content. Consequently, the internet and social media have emerged as a new generation of media tools, playing a significant role in shaping trust in healthcare systems and healthcare professionals [8,22,23,24]. In comparison to conventional media channels, the internet provides a more extensive network of interaction, thereby diversifying the mechanisms that influence the doctor-patient trust relationship and rendering the direction of this relationship uncertain. Firstly, the internet’s increased capacity for disseminating information has facilitated individuals’ access to health-related information, reduced information asymmetry, and enabled patients to adopt more active roles in healthcare processes. This development has led to a redefinition of power dynamics in the patient-physician relationship, and the extant literature emphasizes that the internet’s impact on trust depends not only on the quality of information but also on the communication style physicians exhibit in their interactions with patients. Research indicates that a respectful, supportive, and collaborative approach can mitigate patients’ skepticism and enhance their level of trust [25,26,27,28].

Secondly, the advent of internet technologies has led to a diversification of patient-physician communication channels. Online diagnosis and consultation services have emerged as a complement to traditional face-to-face interactions. Communication processes carried out through online applications and chat modules have become more comprehensive in terms of both frequency and content. The provision of direct access to medical records and information regarding treatment processes by healthcare providers has been demonstrated to enhance patients’ perception of transparency in healthcare services, thereby fostering increased trust in physicians [29,30,31,32].

Thirdly, the pervasive utilization of social media platforms has effectively expanded the boundaries of interaction between physicians and patients. The practice of sharing information on social media platforms and the creation of personal profiles enables patients to evaluate physicians based on criteria that extend beyond professional competence to encompass personal and social aspects. This transformation of the relationship of trust into a more complex and dynamic structure is of significant importance. Furthermore, social media networks have been shown to play a critical role in the formation and maintenance of doctors’ word-of-mouth reputation, thereby strengthening their social credibility [31,33,34]. In this context, the impact of the internet on trust in physicians and the healthcare system is multifaceted, containing both trust-enhancing and skepticism-feeding dynamics. Consequently, multi-level and systematic empirical research is necessary to enhance our understanding of the nature and effects of this relationship [4,8].

Trust in the healthcare system is defined as the degree of confidence individuals have in healthcare institutions, such as hospitals, clinics, and family health centers, where they receive healthcare services. This situation has undergone significant changes in recent years with the development of print and visual media, social media, and the internet [22,23]. The Central Physician Appointment System, widely used in Türkiye, allows patients to schedule appointments online and attend healthcare facilities accordingly [35]. However, due to the nature of healthcare services, delays in appointment times may be permissible depending on the duration of the previous patient’s examination [36,37,38,39]. Furthermore, patients who present directly at healthcare institutions without an appointment through the central physician system undergo examination at appropriate intervals if the physician is available. Patients or their relatives who are unable to accept this situation or who are angry about the delay in obtaining an appointment are known to make statements on social media that damage the reputation of both the healthcare institution and healthcare workers [40]. Such situations have been shown to have a detrimental effect on the trust placed in healthcare personnel and the healthcare system as a whole. The dissemination of such information through visual media may also contribute to declining public trust. Furthermore, these circumstances have been observed to precipitate acts of violence against healthcare professionals [41,42,43,44]. In order to address these issues, relevant institutions are seeking solutions through public service announcements, while health academics are focusing on analyzing behaviors that contribute to trust concerns. In their publications, they posit that enhancing health literacy levels constitutes a solution to such situations that erode trust [45]. Health literacy may buffer misinformation-driven distrust.

Health literacy is defined as the set of skills that an individual possesses, both individually and systemically, in their interactions with health services. Digital health literacy, in contrast, denotes the capacity of individuals to locate, comprehend, appraise, and proficiently utilize health information derived from electronic sources to address or resolve health-related concerns. When evaluated from a public health perspective, health literacy and digital health literacy are directly related to the functioning of the health system and public health. They are currently recognized as important public health issues. This concept encompasses individuals’ ability to effectively access, understand, and use health information, tools, and services. The presence of suboptimal levels of health literacy and digital health literacy has been demonstrated to engender an elevated risk of distrust in the health system, diminished trust in physicians, and unfavorable health outcomes [45,46,47,48]. Consequently, public health experts and health management academics are conducting research on these topics. Moreover, the Ministry of Health endeavors to enhance digital health literacy levels in the digital age by implementing awareness training programs, with the objective of improving public health, increasing individual health awareness, and preventing potential adverse outcomes. Additionally, it endeavors to enhance public cognizance concerning adverse health information, a matter of considerable public health concern in the digital era. Although studies conducted in Türkiye and elsewhere have examined media use, trust in physicians, and distrust in the healthcare system, these issues have generally been considered separately [8,22,24,29,32,33,34]. Evidence remains limited on how specific dimensions of health news perception, including commercial concerns and advertising, consumption promotion, behavioral change, health behavior exploitation, and trust in health news, are simultaneously related to interpersonal trust in physicians and institutional distrust in the healthcare system. This gap is particularly relevant in the Türkiye context, where social media use, health-related media content, and public debates on healthcare services may influence trust-related perceptions [29,46,47,48,49]. Accordingly, this study contributes to the literature by examining health news perception as a multidimensional factor associated with both trust in physicians and distrust in the healthcare system in an adult Türkiye sample. By considering these two trust-related outcomes within the same analytical framework, the study provides an integrated perspective that extends previous national and international research.

**Figure 1:**
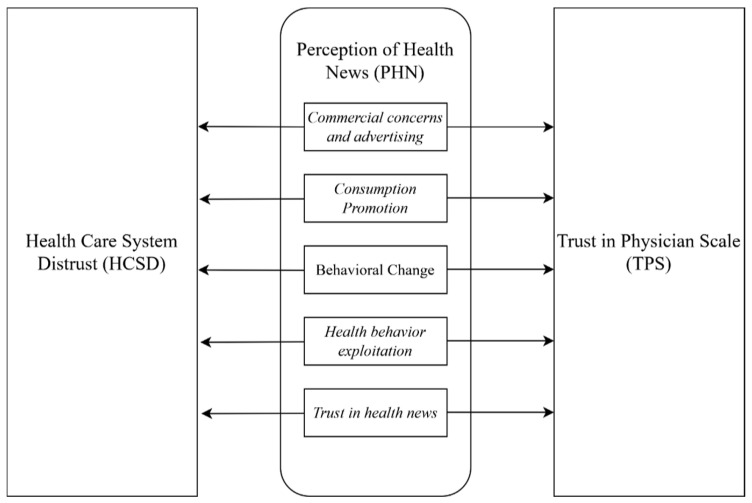
Research Model.

Conceptually, health news perception reflects how individuals interpret the credibility, purpose, and framing of health-related information [18,50]. In this study, health news perception is examined through five subdimensions: commercial concerns and advertising, consumption promotion, behavioral change, health behavior exploitation, and trust in health news. These subdimensions respectively represent perceptions of commercial intent, consumption-oriented messages, behavior-shaping content, emotional or behavioral exploitation, and the perceived credibility of health news. Its subdimensions may shape both evaluations of physicians and broader judgments about the healthcare system by influencing expectations about diagnosis, treatment, and service delivery. Commercially driven, exaggerated, or exploitative health news may increase institutional skepticism and weaken interpersonal trust in physicians [1,4]. Therefore, the model considers health news perception as a multidimensional factor associated with institutional distrust and interpersonal trust. Figure 1 presents the proposed research model.

By addressing both interpersonal and institutional trust, the study adopts an integrated perspective. In this context, the research design developed by the authors is shown in Figure 1.

### Research Hypotheses

**H1.**  *The sub-dimensions of health news perception (commercial concerns and advertising, consumption promotion, behavioral change, health behavior exploitation, and trust in health news) have a positive effect on distrust in the health system.*

**H2.**  *The sub-dimensions of health news perception (commercial concerns and advertising, consumption promotion, behavioral change, health behavior exploitation, and trust in health news) have a negative effect on trust in physicians.*

## 2. Materials and Methods

### 2.1. Study Design and Participants

The present study adopts a cross-sectional research design. The study population consists of 276,427 individuals residing in the central district of Kutahya. The sample size was calculated to be 384, with a 95% confidence interval and a 5% margin of error [51,52,53]. The data were collected in person between December 2024 and March 2025. Data were collected face-to-face from eligible participants in publicly accessible non-clinical areas around hospitals in the central district of Kutahya, such as hospital gardens, cafeterias, and similar social areas. Participants were approached face-to-face in publicly accessible areas of central Kutahya on different days and at different times. The study’s participants were individuals aged 18 to 60 who were literate and consented to partake in the study. Recent use of healthcare services was not an eligibility criterion; it was measured only as a descriptive variable. The age range of 18–60 years was determined in accordance with the predefined scope of the study, which focused on the general adult population residing in the central district of Kutahya. Individuals who met one or more of the following criteria were excluded from the study: refusal to participate, illiteracy, age under 18, or age over 60. The data was collected using the convenience sampling method. Convenience sampling was preferred because probability-based sampling was not feasible due to time, resource, and accessibility constraints during face-to-face data collection. Therefore, individuals who met the inclusion criteria and were accessible during the data collection period were invited to participate in the study. A total of 719 completed survey forms were analyzed in the study.

### 2.2. Data Collection Tools

The study data were collected using a two-part questionnaire prepared in Türkiye. The first section included 16 items on participants’ sociodemographic characteristics and health news experiences. The second section included the Distrust in the Health System, Trust in Physicians, and Health News Perception scales.

Personal Identification Form: The personal information form consists of 11 statements carefully designed by the study authors to elicit information about participants’ sociodemographic characteristics and experiences with health news. This form has not been used in other studies.

Distrust in Health Systems Scale: The scale developed by Rose et al. (2004) [3] was adapted into Türkiye by Yeşildal et al. (2020) [49]. The scale under consideration consists of 10 items and a single dimension. The scale items are presented in a 5-point Likert format, and participants are asked to select the most appropriate option from the range “1-Strongly disagree, 2-Disagree, 3-Undecided, 4-Agree, 5-Strongly agree.” The scale is calculated using the arithmetic mean method. Conversely, elevated scores on the scale are indicative of pervasive distrust in the healthcare system, while diminished scores are associated with minimal distrust. The Cronbach’s alpha value of the study conducted by Yeşildal et al. (2020) was calculated to be 0.78 [3,49]. As indicated in the results section, the Cronbach’s alpha value of the scale in this study was determined to be 0.71.

Trust in Physicians Scale: The scale developed by Anderson and Dedrick (1990) [1] was validated and tested for reliability in Türkiye by Deniz and Çimen (2021) [50]. The scale comprises 11 items. The statements constituting the scale are measured using a 5-point Likert scale (1 = Strongly Disagree, 5 = Strongly Agree). A mean score that approaches 5 indicates a high level of trust in the physician. Items 1, 5, 7, and 11 are negative statements and have been reverse-coded. The Cronbach’s Alpha coefficient of the scale was determined to be 0.90 [1,50]. In this study, the Cronbach’s Alpha value of the scale is 0.76.

Perception of Health News Scale: The scale in question was developed by Çınar et al. in 2018 [47]. The scale is composed of 26 items and 5 subscales: commercial concerns and advertising, consumption promotion, behavioral change, health behavior exploitation, and trust in health news. The statements constituting the scale are measured using a 5-point Likert scale (1 = Strongly Disagree, 5 = Strongly Agree). Each sub-dimension is evaluated independently. Sub-dimension means with an average closer to 5 are indicative of a high perception of the sub-dimension, while means with an average closer to 1 are indicative of a low perception of the sub-dimension. The commercial concerns and advertising dimension encompasses statements pertaining to commercial interests, ratings, and advertising, which may potentially result in the utilization of unnecessary health services. The concept of consumption promotion encompasses a range of strategies aimed at encouraging individuals to procure products or services that were not originally intended to be purchased. These strategies often employ health-related news and programs that suggest potential health risks if the product or service is not used, or alternatively, highlight the purported benefits of the product or service, emphasizing its potential to enhance physical appearance, emotional well-being, and overall health. The second dimension, behavioral change, encompasses health news and programs that encourage the purchase of products or services necessary for treatment, increase the desire to use medication, increase the desire to purchase health products used by famous people, and promote healthy living, diet, cholesterol, cosmetic surgery, cellulite treatment, alternative medicine, medicinal plants, and medicinal stones. The present study explores the relationship between health behavior exploitation and trust in health news. It is hypothesized that there is a correlation between these dimensions and health news and programs that exploit the emotions of patients and their relatives. The Cronbach’s alpha coefficient of the scale was determined to be 0.84 [47]. In this study, the Cronbach’s alpha value of the scale was 0.89.

Scale and subscale scores were calculated as the arithmetic mean of item responses. Negatively worded items in the Trust in Physicians Scale were reverse-coded before score calculation. No weighting procedure was applied. Higher scores indicate greater distrust in the healthcare system, higher trust in physicians, and stronger perception of the relevant health news dimension. Only completed questionnaires were included in the analysis; therefore, no item-level imputation was performed.

### 2.3. Statistical Analysis

Statistical analyses were performed using IBM SPSS Statistics for Windows, version 26.0 (IBM Corp., Armonk, NY, USA). Initially, descriptive statistics, encompassing percentage and frequency analyses, were employed to summarize the demographic characteristics of the participants. Secondly, Pearson correlation analysis was performed to examine the relationships between the scales. To identify the determinants of trust in physicians and distrust in the healthcare system, multiple regression analysis was performed. Multicollinearity among the five health news perception subdimensions was assessed using tolerance and variance inflation factor (VIF) values. Prior to interpreting the Pearson correlation and multiple linear regression analyses, the key statistical assumptions were examined. Normality was assessed using skewness and kurtosis values and visual inspection of residual plots. Linearity and homoscedasticity were evaluated through scatterplots of standardized predicted values and standardized residuals. Influential outliers were examined using standardized residuals, leverage values, and Cook’s distance. The diagnostic checks indicated that the assumptions were adequately met; no serious violation of normality, linearity, homoscedasticity, multicollinearity, or influential outliers was detected.

## 3. Results

Overall, the findings suggest a positive correlation between perceptions of health news and distrust of the healthcare system, and a negative correlation with trust in physicians. The demographic composition of the sample is outlined in Table 1. The majority of the participants, 58%, were aged 41 and above. Furthermore, 57.32% of the participants identified as female, 71% were married, 77.9% were members of a nuclear family, and 71.9% had incomes that did not cover their expenses.

Furthermore, the data indicates that 58.1% of participants seek information from healthcare professionals or hospitals when they experience health problems. The proportion of participants who received healthcare services in the previous month was 87.7%, and 66.5% of them expressed satisfaction with these services. Furthermore, the data indicates that 54.1% of participants utilize social media platforms such as Facebook, Instagram, and TikTok to stay informed about current health news. However, it is noteworthy that only 39.9% of these individuals consistently verify the accuracy of the news they consume, as depicted in Table 2.

The mean score for distrust in the healthcare system is 2.64 ± 0.97 out of 5, the mean score for trust in physicians is 2.98 ± 0.96 out of 5, and the mean score for health news perception is 3.24 ± 0.88 out of 5. For descriptive interpretation, scores between 1.00 and 2.33 were considered low, 2.34–3.67 moderate, and 3.68–5.00 high. The mean values for the subdimensions of health news perception are presented in Table 3.

The mean values range from 3.19 ± 0.89 for commercial concerns and advertising, to 3.17 ± 0.89 for consumption promotion, 3.27 ± 0.83 for behavioral change, 3.20 ± 0.85 for health behavior exploitation, and 3.18 ± 0.86 for trust in health news (Table 3). Based on these cut-off points, participants’ distrust in the healthcare system, trust in physicians, and health news perception were all at moderate levels, although health news perception was above the scale midpoint. Among the health news perception sub-dimensions, behavioral change had the highest mean score, whereas consumption promotion had the lowest mean score. The Cronbach’s alpha coefficients ranged from 0.71 to 0.89, indicating acceptable internal consistency for the scales and subscales. Although all coefficients were within the acceptable range, the alpha values of 0.71 were close to the lower bound. Item–total statistics were reviewed, and no item showed a pattern requiring deletion; however, further studies may examine item-level performance in different samples.

The findings of the correlation analysis revealed a statistically significant positive relationship between distrust in the health system and commercial concerns and advertising, consumption promotion, behavioral change, health behavior exploitation, and trust in health news (r = 0.352, r = 0.309, r = 0.341, r = 0.411, r = 0.339; *p* < 0.01). A negative statistically significant relationship was identified between trust in physicians and commercial concerns and advertising, consumption promotion, behavioral change, health behavior exploitation, and trust in health news (r = −0.397, r = −0.419, r = −0.301, r = −0.429, r = −0.493; *p* < 0.01) (Table 4).

As shown in Table 4, all sub-dimensions of health news perception were positively associated with distrust in the healthcare system and negatively associated with trust in physicians. The strongest positive correlation with distrust in the healthcare system was observed for health behavior exploitation, whereas the strongest negative correlation with trust in physicians was observed for trust in health news. These results suggest that perceptions of health news are linked to both institutional distrust and interpersonal trust in healthcare.

Before conducting Pearson correlation and multiple linear regression analyses, the relevant assumptions were examined. Approximate normality was evaluated using skewness and kurtosis values, with values between −2 and +2 considered acceptable [51]. Linearity and homoscedasticity were assessed through scatterplots of standardized predicted values and standardized residuals, and no serious curved or funnel-shaped pattern was observed [52]. Influential outliers were checked using standardized residuals and Cook’s distance; standardized residuals within ±3 and Cook’s distance values below 1.00 were considered acceptable. Multicollinearity was assessed using tolerance and VIF values; tolerance values above 0.20 and VIF values below 5.00 indicated no serious multicollinearity problem [53]. Overall, the diagnostic results supported the use of the planned analyses.

According to the findings of multiple regression analysis model 1, commercial concerns and advertising, consumption promotion, behavioral change, health behavior exploitation, and trust in health news were positively associated with distrust in the healthcare system in the health system (β = 0.119, β = 0.196, β = 0.054, β = 0.061, β = 0.046; *p* < 0.01, *p* < 0.05). The findings indicate that commercial concerns, advertising, consumption promotion, behavioral change, and trust in health news collectively account for 22.4% of the variation in distrust in the health system (F (5,713) = 41.16; t = 4.761; *p* < 0.01) (Table 5).

Although these associations were statistically significant, the standardized coefficients were generally small (β = 0.046–0.196), indicating that the practical contribution of each individual predictor to distrust in the healthcare system was limited. Among the predictors, consumption promotion showed the relatively strongest positive association with distrust in the healthcare system.

Table 5 shows that the health news perception sub-dimensions explained 22.4% of the variance in distrust in the healthcare system and 25.3% of the variance in trust in physicians. Although the regression coefficients were statistically significant, most standardized coefficients were small, indicating limited practical effects at the individual predictor level. Consumption promotion showed the strongest positive association with distrust in the healthcare system of distrust in the healthcare system, whereas commercial concerns and advertising and trust in health news showed the strongest negative associations with trust in physicians of trust in physicians.

According to the multiple regression analysis model 2, commercial concerns and advertising, consumption incentives, behavior change, misuse of health behaviors, and trust in health news were negatively associated with trust in physicians (β = −0.221, β = −0.014, β = −0.014, β = −0.127, β = −0.211; *p* < 0.05, *p* < 0.01). Commercial concerns and advertising, trust in health news, consumption promotion, behavior change, and exploitation of health behaviors explained 25.3% of the variation in the physician trust scale (F (5,713) = 48.30; t = 14.512; *p* < 0.001) (Table 5). Although statistically significant, the standardized coefficients were small to modest (β = −0.014 to β = −0.221), suggesting that the practical contribution of each predictor to trust in physicians was limited. Commercial concerns and advertising and trust in health news showed the relatively stronger negative associations with trust in physicians.

## 4. Discussion

The present study examined the associations between health news perception sub-dimensions and distrust in the healthcare system and trust in physicians. Given the absence of prior research that has collectively evaluated these three concepts, the findings of this study will be discussed in the context of analogous studies.

The study revealed that 58.1% of the participants sought information from hospitals or health professionals when experiencing a health problem. However, 54.1% of the participants also reported following current health news on social media. The percentage of participants who consistently verified the accuracy of health news they encountered on social media was 39.9%. The proportion of participants who received healthcare services in the previous month was 87.7%, and 66.5% of those participants reported satisfaction with the services received. This percentage suggests that healthcare service satisfaction levels in Türkiye are notably high. A review of extant literature reveals that prior studies on satisfaction with the Türkiye healthcare system have yielded analogous results to those observed in this study [54,55,56]. These findings suggest that, although participants primarily rely on healthcare professionals and hospitals when seeking health-related information, exposure to health news through social media remains an important contextual factor shaping their perceptions of physicians and the healthcare system. In other words, trust in professional sources does not necessarily eliminate the influence of media-based health information on public attitudes. This indicates that professional trust and media exposure may operate simultaneously in shaping perceptions of healthcare.

A study conducted by Özkan et al. (2021) in Türkiye found that 48.6% of participants used the internet as a source of health information [57]. A study conducted by Bach et al. (2024) found that two-thirds of the participants in Germany used the internet to access health-related information [58]. A substantial body of research has indicated that individuals who seek health information online experience an increase in health anxiety and a subsequent development of cyberchondria [59,60]. Furthermore, research has identified a negative correlation between health anxiety, cyberchondria, and digital health literacy [61,62,63]. A study conducted by Dağ et al. (2025) found that as digital literacy levels increase, distrust in the healthcare system and health anxiety decrease [64]. In light of the findings, it can be posited that enhancing digital health literacy in the contemporary digital era has the potential to mitigate distrust in health systems. Indeed, De Gani et al. (2022) have stated that individuals with high health literacy tend to place greater trust in health professionals and public health authorities [65].

The study’s findings indicate that the average perception of health news among the participants (3.24 ± 0.88) was high, while the average levels of distrust in the health system (2.64 ± 0.97) and trust in physicians (2.98 ± 0.96) were found to be moderate. A substantial body of research has been conducted to ascertain the extent of distrust within the Türkiye healthcare system. The findings of these studies have indicated that the level of distrust is moderate [22,23,33,64]. Research conducted by Karaca (2021) and Kaya and Kılıç (2022) has determined that the level of perception regarding health news is high [66,67]. In a study by Çiftçi Kıraç (2024) examining trust in physicians and patient satisfaction, the level of trust in physicians was found to be moderate, similar to the results of the present study [6]. As indicated in the research conducted by Temel and Şantaş (2024) and Ertaş et al. (2025), the level of trust in physicians was found to be high [68,69]. The observed variation in physician trust levels can be attributed to disparities in the socioeconomic profiles of the study participants and the geographical location of the research sites.

While the study identified a negative correlation between trust in physicians and commercial concerns, advertising, consumption promotion, behavioral change, and exploitation of health behavior and trust in health news, it is crucial to acknowledge that this relationship is merely correlational and not causal. This negative relationship may be explained by misinformation, sensationalism, and media framing. Health news emphasizing commercial interests, risks, medical errors, or conflict may shape perceptions of physicians beyond personal healthcare experiences. Repeated exposure to exaggerated or negatively framed content may create unrealistic expectations about diagnosis, treatment, and service delivery. When these expectations are not met, trust in physicians may decrease. Alkan et al. (2022) [28] discovered that as the duration of television viewing increased, there was a concomitant rise in the levels of trust exhibited by participants in the medical decisions made by physicians and the quality of the medical care they received. The aforementioned study revealed a negative correlation between the number of social media accounts possessed by individuals and their confidence in their physicians’ medical decisions [28]. However, no association was observed between the duration of time spent on social media and the aforementioned trust in physicians. However, a contradictory study identified a negative correlation between time spent online and social media use, and trust in medical professionals [8]. Consequently, further research is necessary to ascertain from which media outlets health news is accessed from and to elucidate the individual and societal ramifications of this phenomenon.

The study’s findings indicate that commercial concerns, advertising, consumption promotion, behavioral change, health behavior exploitation, and trust in health news were negatively associated with trust in physicians. As demonstrated in their study, Deniz and Çimen ( 2021) found that trust in physicians increased with age and decreased with education level [50]. Çiftçi Kıraç (2024) found that patient satisfaction increases physician trust [6], while Oguro et al. (2021) determined that dissatisfaction with the health services received by a family member reduces physician trust [11]. Gao et al. (2024) reported that time spent online and frequency of social media use were negatively associated with patients’ trust in physicians [8]. This study, in conjunction with the findings of previous studies, indicates that trust in physicians is influenced by a variety of factors. Consequently, further research is necessary to explore the factors that contribute to this phenomenon.

The study’s findings indicated that distrust in the healthcare system was positively associated with commercial concerns and advertising, consumption promotion, behavioral change, health behavior exploitation, and trust in health news. A study conducted in Türkiye determined that an increase in the level of trust in social media and the amount of time spent on social media was associated with an increase in the behavior of verifying and searching for health news on social media [70]. It has been demonstrated that allocating a substantial portion of one’s time to social media platforms can have a profound impact on one’s life, to the extent that it may be influenced by the virtual environment. Continued exposure to these conditions has the potential to culminate in a significant public health crisis. Consequently, the potential for control exists through the enhancement of social media literacy and digital health literacy within societies [64,71].

A study was conducted to examine the relationship between digital health literacy, health anxiety, and distrust in the healthcare system among university students in Türkiye. The findings indicated that as digital literacy levels increased, health anxiety and distrust in the healthcare system decreased [64]. A multitude of studies have previously concluded that digital health literacy has the potential to mitigate distrust within the healthcare system [33,35,45]. In a study conducted by Çiftçi Kıraç (2024) [6] on the relationship between distrust in the healthcare system and delayed healthcare demand, it was determined that as distrust in the healthcare system increases, healthcare demand is delayed. In the aforementioned study, the level of distrust in the healthcare system among individuals aged 18–24 was found to exceed that of other age groups [6]. The maintenance of good health is an essential service that cannot be postponed. The act of postponing healthcare can result in the exacerbation of preexisting health concerns, leading to prolonged hospital stays and the potential accumulation of additional expenses for social security benefits. The underutilisation of healthcare services by young individuals can be attributed to their increased engagement with digital technologies and social media. Tunç et al. (2025) found in their study that trust in public health authorities reduces distrust in the healthcare system, and that health literacy mediates this relationship [43]. As indicated by the present study and corroborated by extant research, distrust in the healthcare system is influenced by numerous independent variables. The most salient of these factors include health news perception, social media trust level, low health, and digital health literacy, the amount of time spent on social media, and young age. Consequently, it can be posited that enhancing public health literacy, digital health literacy, and social media literacy may contribute to reducing distrust in the healthcare system and supporting public health. Consequently, subsequent studies should employ longitudinal and experimental designs to provide a more precise explanation of causal relationships. Although the models explained 22.4% of the variance in distrust in the healthcare system and 25.3% of the variance in trust in physicians, a considerable proportion of variance remained unexplained. This indicates that unmeasured factors, including previous healthcare experiences, health literacy, physician–patient communication, socioeconomic characteristics, and media exposure patterns, may also contribute to trust-related perceptions.

### 4.1. Limitations

This study has several limitations. First, because of its cross-sectional design, causal inferences cannot be drawn regarding the relationships between perceptions of health news, trust in physicians, and distrust in the healthcare system. Thus, the findings should be interpreted as associations rather than causal effects. It is also possible that the health-related media agenda during the data collection period influenced participants’ perceptions of trust; however, such exposure was not measured, and its potential effect could not be evaluated. Second, the use of convenience sampling may have introduced selection bias and restricted the generalizability of the findings to the wider adult population. Since the study was conducted only in the central district of Kutahya, Türkiye, the results may reflect the sociocultural, geographical, and healthcare system characteristics of this particular setting. For this reason, the findings should be generalized with caution, as results obtained from a single central district may not be transferable to rural areas, other provinces of Türkiye, or countries with different media environments and healthcare system structures.

A further limitation is that adults over 60 years of age and illiterate individuals were not included in the sample. This may limit the applicability of the findings to older populations and may have led to the underrepresentation of groups with lower digital literacy or different patterns of healthcare service use. Since older adults tend to use healthcare services more frequently, their trust-related perceptions may differ from those of younger groups. Future studies should therefore include this age group and examine potential age-based differences. In addition, several possible confounding variables, including socioeconomic status, chronic disease status, previous negative experiences with healthcare services, type of health insurance, health literacy, and intensity of media exposure, were not fully controlled. These factors may affect both perceptions of health news and trust-related attitudes toward physicians and the healthcare system. Furthermore, all variables were measured through self-reported questionnaire data collected at a single time point, which may have introduced self-report and common-method bias. Given the potentially sensitive nature of attitudes toward physicians and the healthcare system in Türkiye, social desirability bias may also have shaped participants’ responses, especially because the data were collected face-to-face. Finally, as multiple correlations and regression coefficients were examined without formal adjustment for multiple testing, the possibility of type I error inflation should be considered. Future research should employ longitudinal or experimental designs to clarify the direction of the observed relationships. Multicenter studies using probability-based sampling, as well as cross-national comparative designs, are also recommended to examine whether the relationship between health news perception and trust in healthcare varies across cultural and health system contexts.

### 4.2. Theoretical and Practical Implications

This study suggests that health-related news is not merely a tool for conveying information, but also creates a field of interpretation that can influence trust in physicians and distrust in the healthcare system. It is observed that trust cannot be considered at a single level; interpersonal trust and institutional distrust can be shaped together within the same information environment. This framework indicates that the concept of trust in public health literature should be considered as a multi-component and multi-level structure. These findings contribute to discussions on institutional trust, misinformation, and infodemic management. Excessive or misleading health information may weaken confidence in health authorities and complicate crisis communication. Thus, health news perception should be viewed not only as an individual response to media content but also as part of a broader information environment that may shape institutional legitimacy and public trust. The theoretical contribution of the study is to demonstrate the explainability of trust relationships through the sub-dimensions of health news perception. It is understood that perceptions of health news carrying commercial concerns and consumption emphasis change along with trust relationships. This situation shows that trust in information and trust in institutions do not always move in the same direction. Thus, trust is considered a social outcome formed not only with the reliability of the source but also with the frame of the message, the implied purpose, and the perception of public benefit.

In practical terms, the findings show that public health communication cannot be reduced to a lack of information. Since perceptions of health news form a decisive basis for trust, the quality of the health information environment is a critical context for maintaining and strengthening trust at the population level. From a policy perspective, the findings indicate the importance of guidelines that support responsible health journalism, including the use of accurate sources, avoidance of sensational language, clear distinction between evidence-based information and commercial content, and careful communication of risks and uncertainties. Partnerships among health authorities, public health experts, and media organizations may also improve the accuracy, clarity, and credibility of health-related news. These collaborations may involve expert review processes, timely correction of misleading information, public evidence summaries, and media literacy initiatives that help the public critically evaluate health news. In this context, the impact of misinformation and misleading frames is assessed not only through individual behaviors but also through the acceptance of health services, interaction with health institutions, and health policy. These implications concern several stakeholders. The Ministry of Health may establish evidence-based communication standards and rapid response mechanisms for misleading health news. Professional associations can support healthcare workers in public communication, while media regulators and journalism schools may strengthen ethical principles in health reporting. Collaboration between media organizations and public health experts may also improve the quality of health information and support trust in physicians and the healthcare system. Literacy-based interventions may be implemented through school curricula, public awareness campaigns, and community-based programs. These interventions may focus on evaluating health news, recognizing misinformation, distinguishing evidence-based information from commercial content, and supporting adults with different literacy and digital access levels. Such approaches may improve critical evaluation of health-related media content and promote informed engagement with healthcare services.

The study also shows that distrust in the health system can be addressed in connection with the news ecosystem. This relationship strengthens discussions on risk communication and institutional legitimacy in public health. Declining trust in physicians provides a meaningful backdrop for broader consequences that could be linked to processes such as delays in service use and reduced treatment adherence. Increased distrust at the institutional level is also an important indicator of the applicability of health policies and the capacity for social solidarity.

Finally, while the cross-sectional nature of the study requires cautious interpretation regarding the direction of relationships, the perception of health news provides a comprehensive assessment in public health literature by revealing the relationship between trust and distrust on the same analytical ground. This comprehensive framework produces an empirical basis explaining the connection between the information environment and the social acceptance of the health system. The Türkiye context may provide relevant insights for other middle-income countries with similar challenges, including centralized appointment systems, growing use of social media for health information, and violence against healthcare workers. In these settings, responsible health communication, digital health literacy, and institutional trust may help sustain public confidence and healthcare service use.

## 5. Conclusions

Based on the observed associations, policies and practices aimed at supporting trust in physicians, reducing distrust in the healthcare system, and improving the quality of health news should be developed. Consequently, given the cross-sectional design, these recommendations should be interpreted as implications based on observed associations rather than causal effects. Educational programs may be designed to enhance public health literacy, digital health literacy, and media literacy. The delivery of these educational programs should be undertaken by health informatics and other health academics, particularly public health specialists at the high school level. Furthermore, the development of diverse communication strategies and social participation methods is imperative to foster enhanced communication and trust between individuals and physicians. Furthermore, healthcare professionals should regularly share information through written and visual media, including social media and television, using clear, transparent, and simple language supported by verifiable data to build trust.

## Figures and Tables

**Table 1:** Socio-demographic characteristics of participants.

Variables	N (719)	%
Age		
≤40	302	42.0
≥41+	417	58.0
Gender		
Female	412	57.3
Male	307	42.7
Education		
High school and below	482	67.1
University	237	32.9
Marital status		
Married	511	71.0
Single	208	29.0
Income Perception		
Income is less than expenses	517	71.9
Income equals expenses	189	26.2
Income exceeds expenses	13	1.9
Family Type		
Extended family	61	8.5
Nuclear family	560	77.9
Single-parent family	98	13.6

**Table 2:** Participants’ health news monitoring behaviors.

Variables	N (719)	%
When seeking information about health issues, which sources do you typically use?		
Medical professionals/Hospitals	418	58.1
Internet/Social media	114	15.8
Television/Radio	106	14.7
Family and friends	81	11.4
When was the last time you received health care?		
Within the last month	630	87.7
Within the last 6 months	89	12.3
How satisfied are you with healthcare services?		
Very satisfied	13	1.9
Satisfied	478	66.5
Undecided	194	26.9
Not satisfied	34	4.7
Where do you get your health news?		
TV	205	28.5
Social media (Instagram, Twitter, etc.)	389	54.1
Health-focused websites	67	9.3
Family/friend	58	8.1
Questioning the accuracy of healthcare news		
Yes, always	287	39.9
Sometimes	364	50.6
Rarely	57	7.9
No, I don’t question it	11	1.6

**Table 3:** Scale and subscale means.

Scales and Sub-Dimensions	Items	Min–Max.	M	*SD.*	Cronbach α
1. Health Care System Distrust (HCSD)	10	1–5	2.64	0.97	0.71
2. Trust in Physician Scale (TPS)	11	1–5	2.98	0.96	0.76
3. Perception of Health News (PHN)	26	1–5	3.24	0.88	0.89
3.1. Commercial concerns and advertising	7	1–5	3.19	0.89	0.84
3.2. Consumption Promotion	3	1–5	3.17	0.89	0.75
3.3. Behavioral Change	7	1–5	3.27	0.83	0.81
3.4. Health behavior exploitation	6	1–5	3.20	0.85	0.71
3.5. Trust in Health News	3	1–5	3.18	0.86	0.77

**Table 4:** Pearson correlation coefficients among study variables.

Scales and Sub-Dimensions		1.	2.	3.	4.	5.	6.
**Model 1 ^a^**	1. Health Care System Distrust (HCSD)	1	0.352 *	0.309 *	0.341 *	0.411 *	0.339 *
	2. Commercial concerns and advertising		1	0.330 **	0.357 *	0.359 **	0.484 **
	3. Consumption promotion			1	0.331 **	0.348 *	0.461 *
	4. Behavioral change				1	0.458 **	0.511 **
	5. Health behavior exploitation					1	0.479 *
	6. Trust in health news						1
**Model 2 ^b^**	1. Trust in Physician Scale (TPS)	1	−0.397 *	−0.419 *	−0.301 *	−0.429 *	−0.493 *
	2. Commercial concerns and advertising		1	0.431 **	0.457 **	0.459 **	0.484 **
	3. Consumption promotion			1	0.331 **	0.448 **	0.461 **
	4. Behavioral change				1	0.436 **	0.411 **
	5. Health behavior exploitation					1	0.418 **
	6. Trust in health news						1

* Correlation is significant at the 0.01 level, ** Correlation is significant at the 0.05 level. ^a^ Correlations with Health Care System Distrust. ^b^ Correlations with Trust in Physicians.

**Table 5:** Multiple regression analyses predicting distrust in the healthcare system and trust in physicians.

Type	Items	B	SH	β	t	*p*
**Model 1 ^a^**	Constant	1.698	0.357		4.756	<0.01
	Commercial concerns and advertising	0.131	0.051	0.119	2.583	<0.01
	Consumption Promotion	0.214	0.073	0.196	2.932	<0.05
	Behavioral Change	0.063	0.032	0.054	1.963	<0.05
	Health behavior exploitation	0.067	0.026	0.061	2.583	<0.01
	Trust in Health News	0.053	0.027	0.046	1.963	<0.05
	**R^2^ = 0.224**	**F = 41.16**		df (5,713)		
	**Dependent Variable:** Health Care System Distrust (HCSD)					
**Model 2 ^b^**	Constant	4.872	0.335		14.512	<0.001
	Commercial concerns and advertising	−0.242	0.067	−0.221	−3.612	<0.001
	Consumption Promotion	−0.015	0.069	−0.008	−1.963	<0.05
	Behavioral Change	−0.016	0.006	−0.014	−2.583	<0.01
	Health Behavior Exploitation	−0.139	0.069	−0.127	−2.017	<0.05
	Trust in Health News	−0.239	0.070	−0.211	−3.414	<0.001
	**R^2^ = 0.253**	**F = 48.30**		df (5,713)		

**Dependent Variable:** ᵃ Dependent variable: Health Care System Distrust; ᵇ Dependent variable: Trust in Physician Scale.

## Data Availability

The datasets used and/or analyzed during the current study are available from the corresponding author upon reasonable request.
